# Complete mitochondrial genome of *Phyllonorycter ringoniella* (Lepidoptera: Gracillariidae)

**DOI:** 10.1080/23802359.2022.2072245

**Published:** 2022-05-10

**Authors:** Nan Zhou, Guoliang Xu, Zhanbao Song, Shengping Zhang, Chun Meng, Dezhi Lv, Sumiao Yang

**Affiliations:** a Shijiazhuang Institute of Pomology, Hebei Academy of Agriculture and Forestry Sciences, Shijiazhuang, Hebei, P. R. China; b Pingquan Agricultural and Rural Bureau, Chengde, Hebei, P. R. China; c Shenzhou Liangsang Breeding Farm, Hengshui, Hebei, P. R. China

**Keywords:** Mitochondrial genome, Gracillariidae, *Phyllonorycter ringoniella*, phylogenetic analysis

## Abstract

The complete mitochondrial genome of *Phyllonorycter ringoniella* (Matsumura, 1931) is characterized by a circular with 15,729 bp in size, containing 37 encoded genes and a control region. The gene order and nucleotide composition are similar to the known gracillariid mitogenomes. All protein-coding genes (PCGs) initiate with ATN and terminate with TAN, while *cox1* starts with CGA, *cox1, cox2, nad3,* and *nad5* terminate with an incomplete codon TA– or T–. All transfer RNA genes (tRNAs) can fold into typical clover-leaf structure, except for *trnS1* (AGN), in which dihydrouridine stem is simplified to form a loop structure. The control region is located between *12S rRNA* and *trnM* with relatively strong AT bias. The phylogenetic trees reveal that two subfamilies Oecophyllembiinae and Acrocercopinae were clustered together, and that clade was sister to the subfamily Lithocolletinae. All species of the genus *Phyllonorycter* grouped into a monophyletic clade and the *P. ringoniella* was closely related to *P. platani*.

The apple leafminer, *Phyllonorycter ringoniella* (Matsumura, 1931) (Gracillariidae: Lithocolletinae) (Kumata 1963), is a serious and common pest of many poem and stone fruits including apple, pear, peach, cherry, and plum in major apple-growing regions of Russia, Korea, Japan, and China (Lee et al. 1985; Li et al. 2002; Sun et al. 2007; Du et al. 2013; Kirichenko et al. 2017). In its earlier stages, the larvae feeds on the spongy mesophyll cell and create mines with silver-green spot of irregular shape on the lower surface of the leaf. As the larvae develops, it feeds toward the upper surface chewing out palisade cells as far as epidermis on the upper side, which caused green-white appearance on the upper surface of the leaf (Sekita and Yamada 1979). The damages caused by *P. ringoniella* decrease the photosynthetic area, hasten defoliation, and inhibit the growth of new buds, which may finally cause premature ripening and fruit drop (Sugie et al. 1986; Boo and Jung 1998; Shi et al. 2009).

Herein, the complete mitogenome of *P. ringoniella* was sequenced. The phylogenetic relationship was inferred based on published mitogenome sequences of gracillariids in GenBank with this newly sequenced mitochondrial genome of *P. ringoniella* in this study. Adult specimens of *P. ringoniella* were collected by pheromone traps in October, 2021 from an apple orchard, Changan district, Shijiazhuang City, Hebei Province, China (38°12′N, 114°52′E). Specimens were preserved in pure ethanol in a −20 °C freezer. The voucher specimen (Voucher number SGSGRA01) was stored in the insect collection, Shijiazhuang Institute of Pomology (URL: http://www.hebnkysgs.com/; Contact person: Sumiao Yang, ysm613@126.com), Hebei Academy of Agriculture and Forestry Sciences, Hebei Province, China. Genomic DNA was extracted from thoracic muscles and sequenced using the Illumina NovaSeq platform with paired-end reads of 2 × 150 bp at Personalbio Technology Co. Ltd., (Shanghai, China). MitoZ version 2.4 was performed to construct annotated mitochondrial genes from raw data under *all* module by default (Meng et al. 2019). The annotated mitogenome was rechecked using Geneious version 11.0.2 (Biomatters, Auckland, New Zealand), with the published mitogenomes of gracillariids as references before data analyses.

The complete mitogenome of *P. ringoniella* (GenBank accession number. OM287125) was characterized by a circular molecular structure of 15,729 bp in length, containing 13 PCGs, 22 transfer RNAs, 2 ribosomal RNAs, and a control region, with similar gene arrangement and content to those of most other moths. The nucleotide composition was 40.9% A, 40.6% T, 11.1% C, 7.5% G, with slightly positive AT-skew and negative GC-skew. Four overlapping regions (ranging from 1 bp to 8 bp) and 23 intergenic spacer regions (ranging from 1 to 151 bp) were found in the whole mitogenome. All PCGs encoded 3,726 amino acids with total length of 11,178 bp, four genes (*nad1*, *nad4*, *nad4L*, and *nad5*) were in L-strand and the remaining genes were in H-strand. All PCGs initiated with start codon ATN and terminated with stop codon TAN, except *cox1* started with CGA, *cox1, cox2, nad3,* and *nad5* stop with an incomplete codon TA- or T–. The secondary structures of 22 tRNAs exhibited cloverleaf structure except *trnS1* (AGN) with dihydrouridine (DHU) arm lost. The length of *12S rRNA* and *16S rRNA* were 766 bp and 1,486 bp, respectively. The control region was located between *12S rRNA* and *trnM* with relatively strong AT bias.

To validate the phylogenetic position of *P*. *ringoniella*, the mitogenomes of 7 species within Gracillariidae were selected as ingroups (Timmermans et al. 2014; Chen et al. 2019; Lu et al. 2019; Liu et al. 2020; Zhang et al. 2020a) and *Tineola bisselliella* (Hummel, 1823) (Tineidae: Tineinae) was selected as outgroup (Timmermans et al. 2014). Phylogenetic analyses were performed by maximum likelihood (ML) and Bayesian inference (BI) methods based on the concatenated nucleotide sequences of 13 PCGs. The alignment of all 13 PCGs was conducted in batches using codon alignment mode by MAFFT version 7.313 plugin in PhyloSuite version 1.2.1 (Katoh and Standley 2013; Zhang et al. 2020b). The ML analysis was implemented by IQ-TREE version 1.6.8 under ultrafast bootstrap with 1000 replicates. The BI analysis was implemented by MrBayes version 3.2.6 with default settings and 5 million generations, sampling per 1000 generations (Ronquist et al. 2012; Nguyen et al. 2015). The best partitioning schemes and substitution models for ML and BI analyses were estimated by PartitionFinder version 2.1.1 (Lanfear et al. 2016). Both ML and BI analyses yielded a consensus topology (Figure 1). The results indicated that two subfamilies Oecophyllembiinae and Acrocercopinae were clustered together, and that clade was sister to the subfamily Lithocolletinae. All species of the genus Phyllonorycter grouped into a monophyletic clade and the *P. ringoniella* was closely related to *P. platani*.

**Figure 1.**
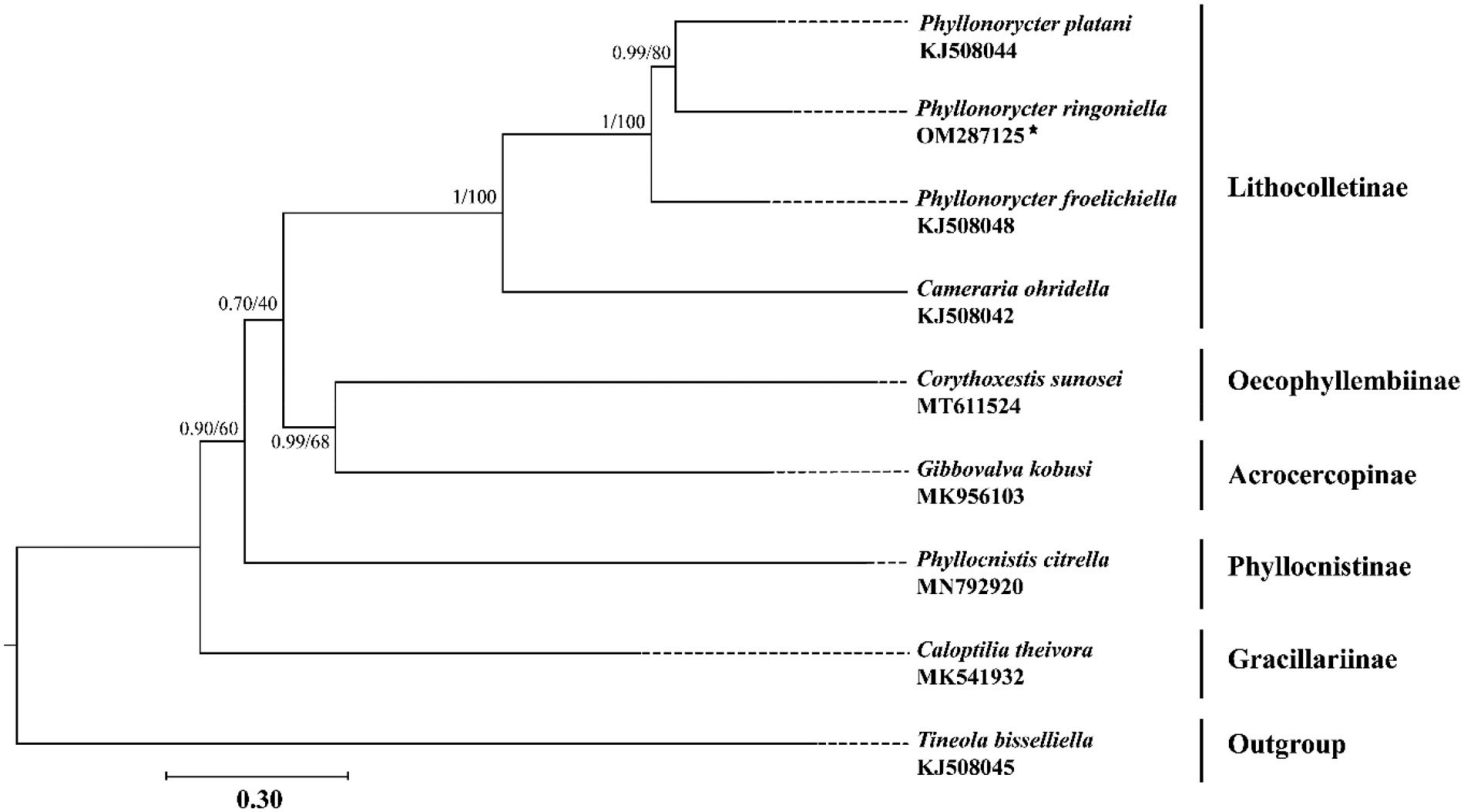
Phylogenetic tree using maximum likelihood (ML) analyses based on concatenated nucleotide sequences of 13 PCGs. Bayesian inference (BI) analyses show the same topology (not shown). The numbers under the branches are Bayesian posterior probabilities and bootstrap support values. Alphanumeric terms indicate the GenBank accession numbers.

## Data Availability

The genome sequence data that support the findings of this study are openly available in GenBank of NCBI at https://www.ncbi.nlm.nih.gov under the accession number OM287125. The associated BioProject, SRA, and BioSample accession number are PRJNA797163, SRR17617338, and SAMN24966706 respectively.
